# Scalable Biofabrication of Functional 3D Scaffolds via Synergy of Autopilot Single-Jet Electrospun 3D PCL Fiber Scaffolds and Cell-Laden Hydrogels

**DOI:** 10.1021/acsami.5c07425

**Published:** 2025-07-22

**Authors:** Balchandar Navaneethan, Mehdi Salar Amoli, Yen-Ching Yang, Sarah Rezapourdamanab, Chiao-Yu Tseng, Yamini Singh, Chin-Lin Guo, Vahid Serpooshan, Chia-Fu Chou

**Affiliations:** † Institute of Physics, Academia Sinica, Taipei 11529, Taiwan, ROC; ‡ Biomedical Translational Research Center, National Biotechnology Research Park, 38017 Academia Sinica, Taipei 11571, Taiwan, ROC; § Wallace H. Coulter Department of Biomedical Engineering, Emory University School of Medicine and Georgia Institute of Technology, Atlanta, Georgia 30322, United States; ∥ Department of Pediatrics, Emory University School of Medicine, Atlanta, Georgia 30322, United States; ⊥ Children’s Healthcare of Atlanta, Atlanta, Georgia 30322, United States; # Research Center for Applied Sciences, Academia Sinica, Taipei 11529, Taiwan, ROC

**Keywords:** Biofabrication, 3D bioprinting, electrospinning, functional constructs, cell-laden hydrogels, PCL fiber

## Abstract

3D bioprinting enables cell-laden hydrogel construct fabrication in a layer-by-layer fashion but faces scalability challenges due to the mechanical weakness of hydrogels. Matrix reinforcement compromises cellular activity, creating a scalability-functionality trade-off that remains unresolved as sophisticated strategies including sequential and embedded printing fail to effectively overcome these limitations. This study presents an alternative approach by integrating autopilot single-jet electrospun (AJ-3D ES) 3D PCL fiber scaffolds with hydrogels, achieving anatomical precision, mechanical robustness, and enhanced cell function. Hydrogel dip-coating of anatomically structured PCL scaffolds enabled organ-scale cellular constructs. By providing an ECM-mimicking porous fiber network, embedded cells mitigated the limitations of hydrogel stiffness (even ∼50 kPa) and facilitated cell–cell interactions, supporting epithelialization, fibroblast clustering, and 3D phase-separated HepG2-HUVEC co-cultures. Contour 3D bioprinting along PCL fiber scaffold topographies facilitated endothelial patterning for vascularization and native-tissue mimicking complexity. Volumetric scalability was demonstrated through hydrogel casting, embedded bioprinting, and modular stacking within 3D PCL fiber scaffolds, ensuring hydrogel integrity while maintaining medium diffusion for sustained cell survival and function. In vivo studies confirmed the proangiogenic nature of PCL fiber scaffolds with tissue bridging via cell infiltration and ECM collagen deposition, underscoring clinical translational potential. By integrating topographic and volumetric flexibility, this approach advances biofabrication strategies for functional tissue and organ constructs.

## Graphical abstract

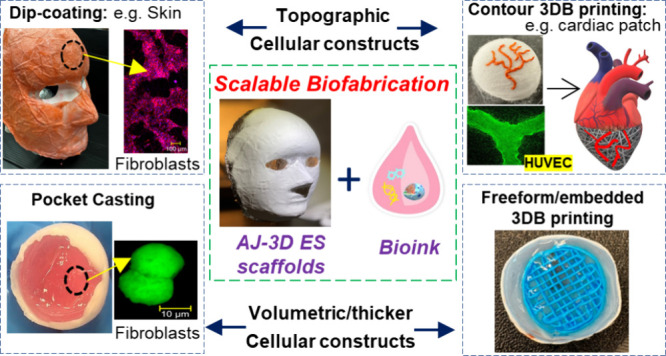

## Introduction

Over the past three decades, advances in tissue engineering and regenerative medicine (TERM) have been marked by increasingly sophisticated technologies.^1–3^ Initial efforts centered around constructs with simple geometries and composition for applications such as wound healing.^4,5^ These evolved into more complex strategies, including three-dimensional bioprinting (3DB printing), which enables the fabrication of cellular constructs by printing cell-laden bioinks followed by physical or chemical hydrogel cross-linking.^6,7^ Various printing modalities have been employed based on application needs. Inkjet printing, which dispenses low-viscosity bioinks in droplet form, offers high cell viability but suffers from poor printing fidelity and limited scalability.^8^ Dynamic interface volumetric printing—also known as two-photon or laser-based cross-linking—achieves high-resolution features (∼tens of microns), though confined to millimeter-scale structures due to optical constraints such as bioink transparency and refractive index.^9,10^ In contrast, extrusion-based bioprinting, which uses high-viscosity bioinks to form continuous filaments, has emerged as the preferred approach for scaffold-based TERM due to its improved printing fidelity, vertical stacking ability, and scalability.^6,11,12^ However, substantial shear forces generated during printing and their dense matrix reported with reduced cell viability and limited cellular function in postprinted constructs demanding prolonged incubation.^13,14^ Conversely, low viscosity bioinks improving cellular function contribute to poor scalability with simultaneous layer-by-layer (LbL) printing and cross-linking approach compromise interlayer structural integrity, rapid degradation, thereby restricting to lab-scale constructs with simple geometries.^15–17^ To address these cell functionality and scalability trade-off limitations, alternative approaches such as sequential printing and freeform and embedded printing have been explored. Sequential printing incorporates thermoplastics, e.g., PCL, as 3D support structures via fused deposition modeling (FDM), offering stability between cell-laden layers, e.g., human ear cartilage, mandible bone,^18–20^ while freeform printing employs supportive baths to stabilize un-cross-linked layers during printing, enabling the use of low viscosity bioinks for the creation of complex, curvilinear structures, e.g., neonatal heart.^21–25^

Despite these innovations, significant challenges persist. Integrating rigid thermoplastic PCL layers, produced via FDM, with hydrogels remains challenging due to their contrasting mechanical properties. The stiffness of PCL layers hampers seamless integration with hydrogels, which lack the flexibility needed to replicate soft tissue behavior effectively. Additionally, the plastic surfaces of PCL fail to promote adequate cell attachment, and the slow degradation of bulk PCL fibers (>100 μm) delays tissue development and extracellular matrix (ECM) remodeling.^26–28^ Freeform constructs, in contrast, depend on the intrinsic stiffness of hydrogels to maintain anatomical shapes after cross-linking. However, high hydrogel stiffness (several 10s to 100s of kPa) reduces mechanical flexibility and compromises cell survival and functionality by impairing cell mobility. This limitation hinders the essential cell–cell interactions required for autonomous self-assembly, tissue formation, and vascular integration. Consequently, demonstrations of tissue and organ printing, such as vascular structures and heart and lung models, remain restricted to smaller, millimeter-scale constructs.^22–24,29,30^ While conventional electrospun fibers are flexible and enhance cell alignment, they have been limited to simple stacking (e.g., skin bilayers—dermis and epidermis) or blending with hydrogels as a rheological modifier,^31–34^ primarily because traditional electrospinning produces 2D sheets rather than 3D structures, resulting in poor scalability and loss of structural integrity as the hydrogel degrades.

To address these challenges, we propose the autopilot single-jet 3D electrospinning (AJ-3D ES) technique,^35,36^ which fabricates 3D PCL fiber scaffolds with versatile geometries, including anatomically accurate topographic structures (Figure 1a). This is achieved through single jet high-fidelity surface topography replication of intricate, mm- to organ-scale templates—an advancement over conventional far-field multijet or near-field single-jet electrospinning approaches.^37–40^ Unlike fragile electrospun scaffolds that deform or sag and rigid bulk-printed PCL constructs, these scaffolds combine mechanical robustness, flexibility, and shape retention, enabled by their fused buckled fiber morphology (Figure S1 and Video S1). In this study, we demonstrate how the innovative AJ-3D ES technique and unique properties of AJ-3D electrospun PCL fiber scaffolds enable a seamless, advanced integration with cell-laden hydrogels to create human-scale constructs. This integration also eliminates the need for excessive hydrogel cross-linking to maintain structural stability, effectively addressing the common trade-off between scalability, flexibility, and functionality in cell-laden hydrogel constructs for tissue engineering (TE).

**Figure 1:**
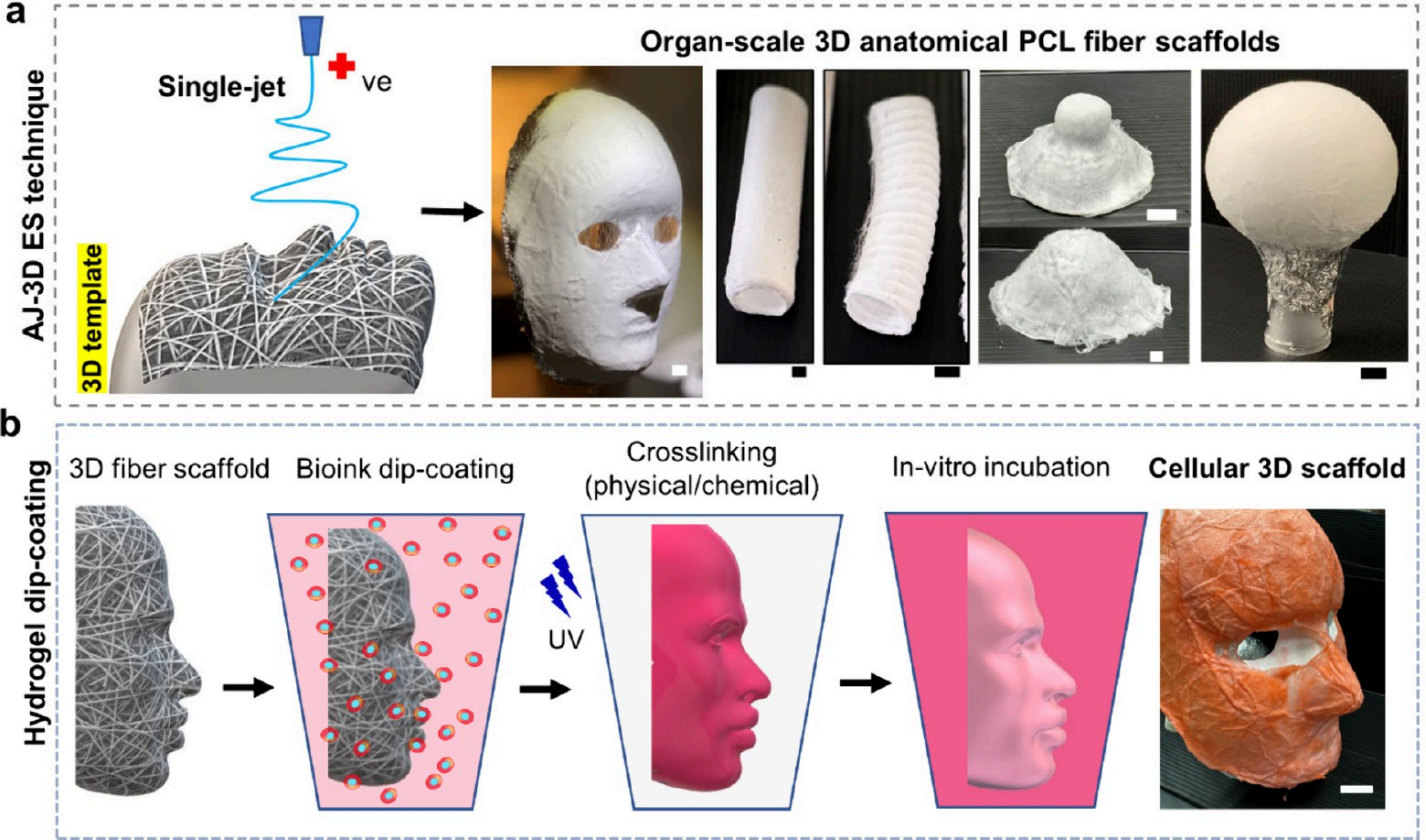
**Biofabrication of cellular anatomical constructs.** (a) Schematic (left) illustrating the single-jet fabrication of anatomically structured 3D scaffolds via template-guided conformal replication using the AJ-3D ES technique alongside (right) representative anatomical geometries, including a human face, blood vessel (tubular), trachea (with rings), nipple, and breast (domes). Scale bar: 1 cm. (b) Schematic depicting the hydrogel dip-coating process: A human facial 3D PCL scaffold is coated with cell-laden bioink, cross-linked, and incubated for tissue development. (Right) SA-dip-coated human facial construct.

## Results

### Hydrogel Dip-Coating for Cell-Embedded, Anatomically Relevant Topographical Constructs

Hydrogel dip-coating of 3D fiber templates with bioinks, comprising hydrogel precursors and cells, conforms seamlessly to the fiber template’s intricate shape during cross-linking, yielding cell-embedded human organ-scale constructs (Figure 1b). Sodium alginate (SA) dip-coated constructs, designed to replicate the topography of a human face, demonstrate a pliable and elastic texture, closely resembling the softness and resilience of native skin (Video S2).

Since the AJ-3D electrospun PCL scaffolds demonstrate the same stiffness across all shapes and sizes, we used circular PCL fiber disks (1.5 cm) here to access cell functionality within these constructs for culture and imaging convenience. These disks were immersed in 1% SA bioink containing Madin-Darby Canine Kidney (MDCK) epithelial cells at a concentration of 1 × 10^6^ cells/mL. The SA bioink absorbed into the spongy PCL fiber templates was then cross-linked by immersion in a CaCl_2_ solution (Figure S2). MDCK cells, known for their ability to form polarized monolayer sheets with apico-basal polarity,^41^ served as an ideal model to assess the scaffold’s capacity to support physiologically relevant tissue architecture (Figure 2a).

**Figure 2:**
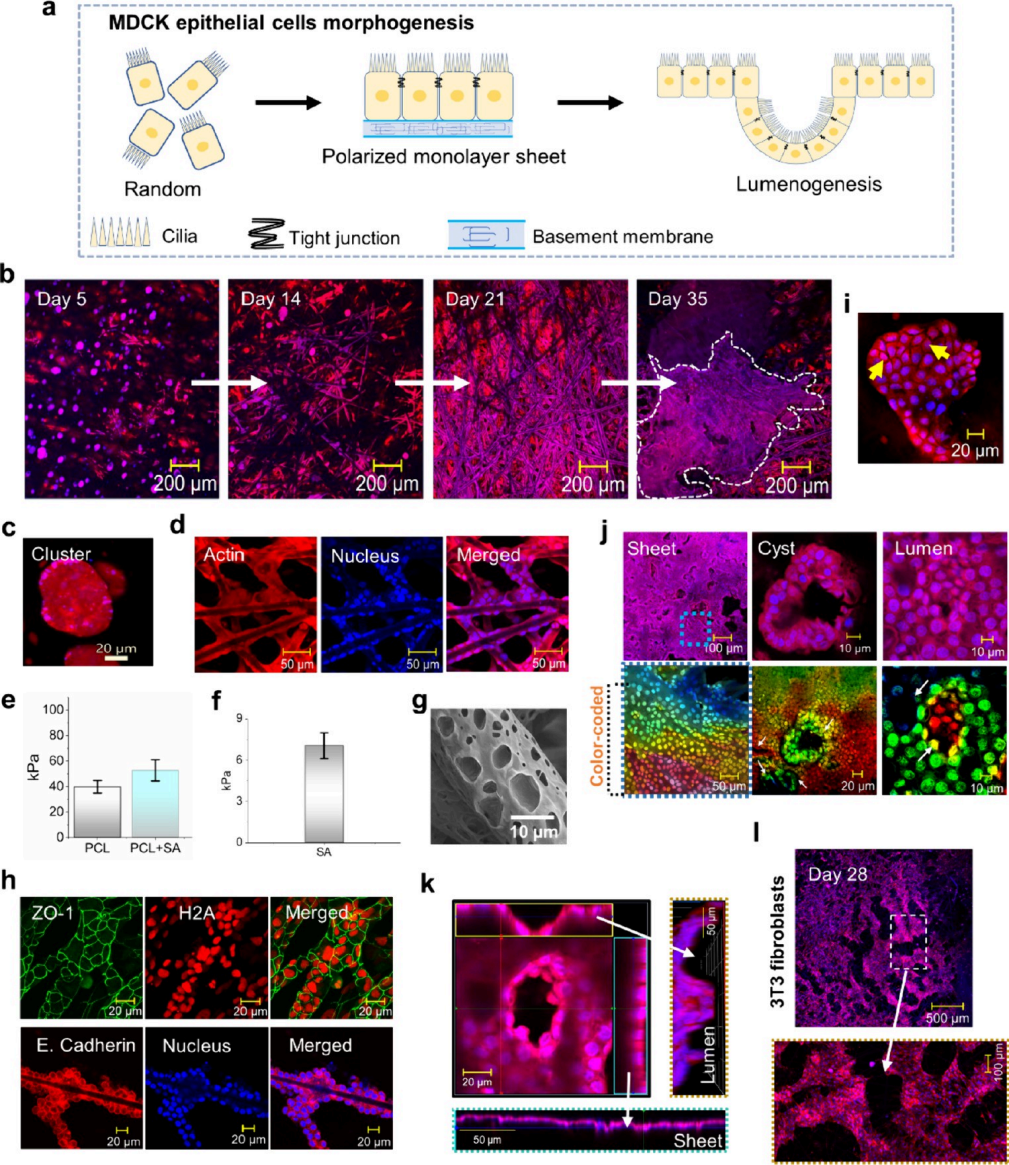
**Autonomous MDCK and fibroblast self-assembly in SA dip-coated constructs.** (a) Schematic of MDCK cell morphogenesis, from monolayer to lumenogenesis. (b) Confocal images of MDCK cells in dip-coated constructs over 35 days with actin (red) and nuclei (blue). The white dotted line encircles the MDCK sheet at day 35. (c, d) Cell morphology in hydrogel (c) and on fibers (d). (e, f) Bar graphs of Young’s modulus from microindentation of PCL constructs: PCL and PCL+SA (e) and 1% SA hydrogel (f), in kPa. (g) SEM of PCL fiber surface pores. (h) Live cell imaging showing tight junction protein ZO-1 (green), H2A histone (red), epithelial cadherin (E-cadherin, red), and nuclei (blue). (i) Confocal image at day 35 showing polygonal epithelial cells with tight junctions (yellow arrows). (j) MDCK cell morphogenesis, with cyst- and lumen-like structures, shown in color-coded projections. (k) Confocal orthographic view showing continuity of the monolayer and presence of a cell tube. (l) Confocal image at day 28 showing 3T3 fibroblast clusters in 1% SA dip-coated constructs. The magnified image below highlights the area in the yellow box.

### Formation of Polarized, Epithelial Tissues and Fibroblast Clusters via Cell Self-Assembly and ECM Remodeling

Confocal imaging over a 35-day incubation period captured diverse cellular activities, including proliferation, migration, and organization within the dip-coated constructs (Figure 2b). By day 5, cells were uniformly distributed across the constructs, with 3D depth profile analysis revealing deeper cell penetration into the fiber networks (Video S3). Cells embedded within the SA hydrogel in fibrous pockets formed compact clusters (Figure 2c). In contrast, cells attached to the PCL fibers exhibited distinct spreading behaviors, elongating along and wrapping around the fibers (Figure 2d).

Microindentation analysis revealed that hydrogel-coated constructs exhibited a stiffness of 52.7 ± 8.2 kPa, higher than that of plain PCL scaffolds, 39.7 ± 4.9 kPa, and the SA hydrogel alone, 7 kPa (Figure 2e,f). Scanning electron microscopy (SEM) revealed the high surface roughness of the PCL fibers, attributed to solvent-induced phase separation, which enhanced cell-fiber interactions (Figure 2g). This traction-mediated attachment significantly promoted cell proliferation and migration, resulting in nearly complete cell coverage of the fibrous networks by day 21 (Figure 2b). Conversely, cells encapsulated within the SA hydrogel were constrained by its stiffness and the absence of morphological cues (Figure 2c), highlighting the limitations of hydrogel-based systems in supporting extensive cell distribution and growth.^42,43^

To investigate MDCK epithelial cell morphogenesis, we examined the apical expression of the tight junction protein Zonula occludens (ZO-1) and epithelial cadherin, critical markers of epithelial tissue development, in GFP-transfected cells adhered to PCL fibers. Confocal imaging revealed well-defined tight junctions between adjacent cells (Figure 2h, upper panels) and the peripheral localization of epithelial cadherin (Figure 2i, lower panels), confirming cell–cell adhesion and contact. By day 35, continuous cellular proliferation resulted in the formation of tissue-like epithelial sheets extending several millimeters (Figure 2b). These sheets comprised polygonal cells with intercellular barriers, signifying the establishment of tight junctions (Figure 2i).

Depth-profile analysis and imaging further revealed cyst-like microstructures embedded within the sheets, with lumens forming in depressions of the monolayer (Figure 2j,k and Video S4). In a parallel experiment, PCL fiber disks seeded with mouse embryonic 3T3 fibroblasts formed large, interconnected spindle-shaped 3D clusters over 28 days (Figure 2l and Figure S3). These observations suggest a dynamic morphogenetic process wherein MDCK cells initially attach to the fibers, proliferate, and migrate. Upon reaching confluency, cell–cell interactions facilitated the collective reassembly, where the migration of MDCK cells toward the top surface resulted in the formation of anisotropic, polarized tight epithelial monolayered sheets. In contrast, 3T3 fibroblasts reassembled into large spindle-shaped clusters within the isotropic random fiber network. The open, interconnected, cell-permeable porous architecture of the PCL fiber networks, in contrast to the tightly packed structure of conventional scaffolds, promoted cell–cell interactions, thus forming large 3D cellular structures across the constructs.

### Co-Cultured HepG2 and HUVECs Exhibit 3D Phase Separation in GelMA Dip-Coated Constructs

To evaluate the potential of hybrid constructs in fostering multicellular interactions under physiological conditions, we conducted a co-culture experiment using a liver tissue model (Figure 3a).^44^ For this, a 5% (w/v) gelatin methacrylate (GelMA) bioink was prepared, incorporating human umbilical vein endothelial cells (HUVECs) and human hepatocellular carcinoma cells (HepG2s) at a 1:5 ratio and high cell densities (2 × 10^6^ HUVECs and 1 × 10^7^ HepG2s per mL). The GelMA bioink, enriched with RGD sequences,^45^ was chemically modified with methacrylate groups to enable UV-based cross-linking (Figure 3b). This modification not only facilitated cross-linking but also enhanced cell-matrix interactions by providing integrin-binding sites, which are absent in SA.^46^

**Figure 3:**
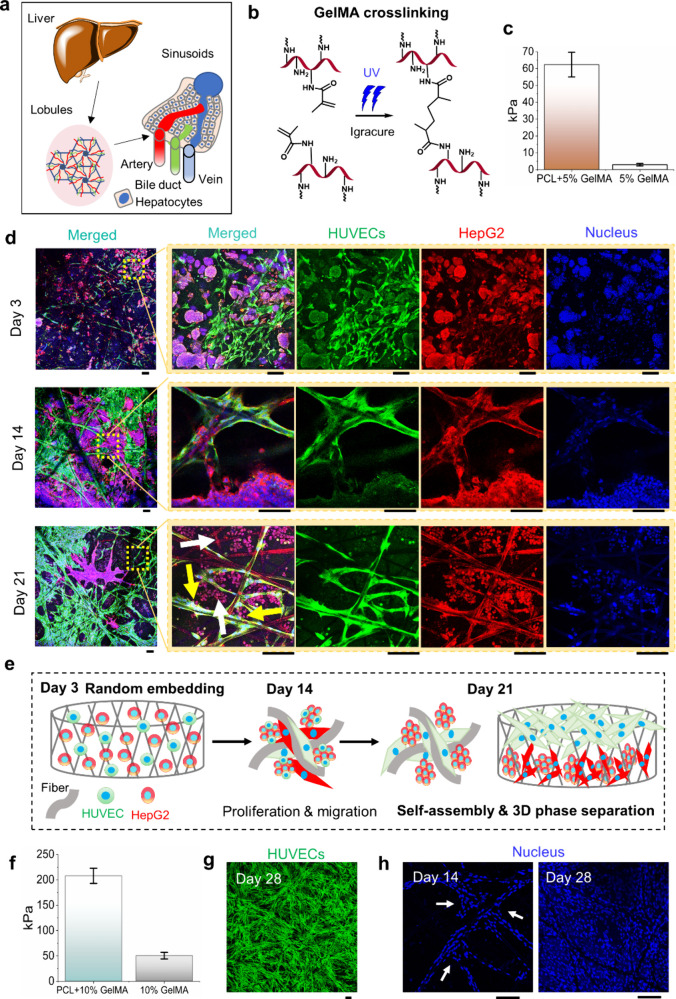
**3D phase separation of co-cultured HepG2 and HUVECs in GelMA dip-coated constructs and the effect of strong GelMA cross-linking.** (a) Schematic of the liver anatomy, showing lobules, sinusoids, and hepatocytes. (b) Reaction scheme for UV cross-linking of GelMA with Irgacure 2959. (c) Bar chart of Young’s modulus from microindentation of 5% GelMA hybrid constructs (PCL+5% GelMA) and 5% GelMA hydrogel. (d) Confocal images showing HUVEC and HepG2 morphology and distribution over 3, 14, and 21 days, with HUVECs (red), HepG2s (green), and nuclei (blue). Yellow and white arrows indicate HUVECs on the top fiber plane and HepG2s beneath. (e) Graphical images illustrating co-cultured cell self-assembly, from random distribution to liver-like organization. (f) Bar chart of Young’s modulus from microindentation of heavily cross-linked 10% GelMA hybrid constructs (PCL+10% GelMA) and 10% GelMA hydrogel. (g) Live cell imaging on day 28 showing the HUVEC distribution in 10% GelMA hybrid constructs. (h) DAPI-stained confocal images of the cell nuclei distribution in 10% GelMA hybrid constructs on days 14 and 28. Scale bar: 100 μm.

The dip-coated constructs exhibited a stiffness of 63 ± 7.3 kPa, while the GelMA hydrogel alone had a stiffness of 3 ± 0.7 kPa (Figure 3c), within the physiological stiffness range of healthy human liver between 0.5 and 6 kPa.^47^ Cellular distribution and morphology were analyzed on days 3, 14, and 21 (Figure 3d). By day 3, cells had migrated across the constructs, forming heterogeneous (both HUVECs and HepG2s) clusters within the fibrous pockets. By day 14, the constructs reached high confluency and phase separation was evident, with distinct spatial organization emerging. By day 21, HUVECs predominantly localized on the top fiber plane, segregating from HepG2s, forming distinct spatial domains within the constructs. HepG2 cells, known for their tumorigenic properties and tendency to form clusters,^48^ progressively detached from the fibers and reorganized into compact, homogeneous clusters within the fibrous pockets beneath HUVECs (Figure 3e). These findings demonstrate the constructs’ ability to support high-order cellular organization, mimicking the dynamic multicellular arrangements seen in native tissues.

### Effect of Stronger GelMA Cross-Linking on Cell Self-Assembly and Tissue Formation

To investigate whether hydrogel degradation or cell permeability primarily drives cellular bridging between fibers, facilitating either extensive rearrangement or segregation, we formulated a HUVEC-suspended bioink with a higher GelMA concentration (10% w/v). Enhanced UV intensity and exposure time were applied to achieve stronger cross-linking and prolonged hydrogel stability. This modification produced hybrid constructs with significantly higher stiffness of 207 ± 14.9 kPa, over three times that of the 5% GelMA constructs, while the 10% GelMA hydrogel alone exhibited a stiffness of 50 ± 6.4 kPa (Figure 3f). Despite the increased matrix stiffness, robust cell proliferation was observed by day 28, indicating that the PCL fibers continued to facilitate cellular activities. Cellular arrangements differed from those in the 5% GelMA constructs, with cells aligning along the fiber axis in the stiffer 10% GelMA constructs (Figure 3g). High-magnification DAPI staining on day 14 (Figure 3h) revealed radial stacking of cells at interfiber junctions, suggesting that these regions act as nucleation sites, promoting cell spreading into the hydrogel matrix. These findings demonstrate that PCL fibers enable tissue formation without needing hydrogel degradation cell migration even in high-stiffness hydrogels, though excessive stiffness may delay cell bridging across the matrix.

### Influence of Hydrogel Stiffness on Dip-Coated Constructs

Tensile strength testing on dip-coated constructs using 1% SA, 5% GelMA, and 10% GelMA was performed to assess the influence of hydrogel integration on the bulk mechanical properties of fiber-hydrogel composites in comparison to PCL alone (Figure S4). The results showed that all dip-coated constructs exhibited similar stress–strain trends to pure PCL and reached comparable ultimate tensile strengths (UTS) in the range of ∼0.69–0.84 MPa, at high strains between 1102 and 1284%. These values were not significantly different from pure PCL, which showed a UTS of 0.89 MPa at 1088% strain. However, the dip-coated samples exhibited a broader and lower range of Young’s modulus values of 0.96 to 4.81 MPa compared to pure PCL, indicating that hydrogel integration primarily affects the initial stiffness without compromising the overall strength or extensibility of the constructs.

### Contour 3DB Printing on PCL Fiber Templates for Vascularized Tissue Constructs

While hydrogel dip-coated constructs facilitate the self-assembly of randomly embedded cells, 3DB printing enables systematic cell deposition along the intricate topography of fiber templates. This approach supports the fabrication of complex, microscopic tissue- and organ-mimicking 3D anatomical constructs and allows for the printing of endothelial cells, which enhance vascular integration by promoting blood vessel formation, critical for constructing in vivo survival and functionality (Figure 4a). To demonstrate this feasibility, we used an in situ 3DB printer (Bioassembly Bot 500) to print test inks onto fiber templates (Figure 4b). Unlike conventional 3DB printers, in situ systems offer enhanced flexibility, enabling accurate printing on nonflat surfaces.

**Figure 4:**
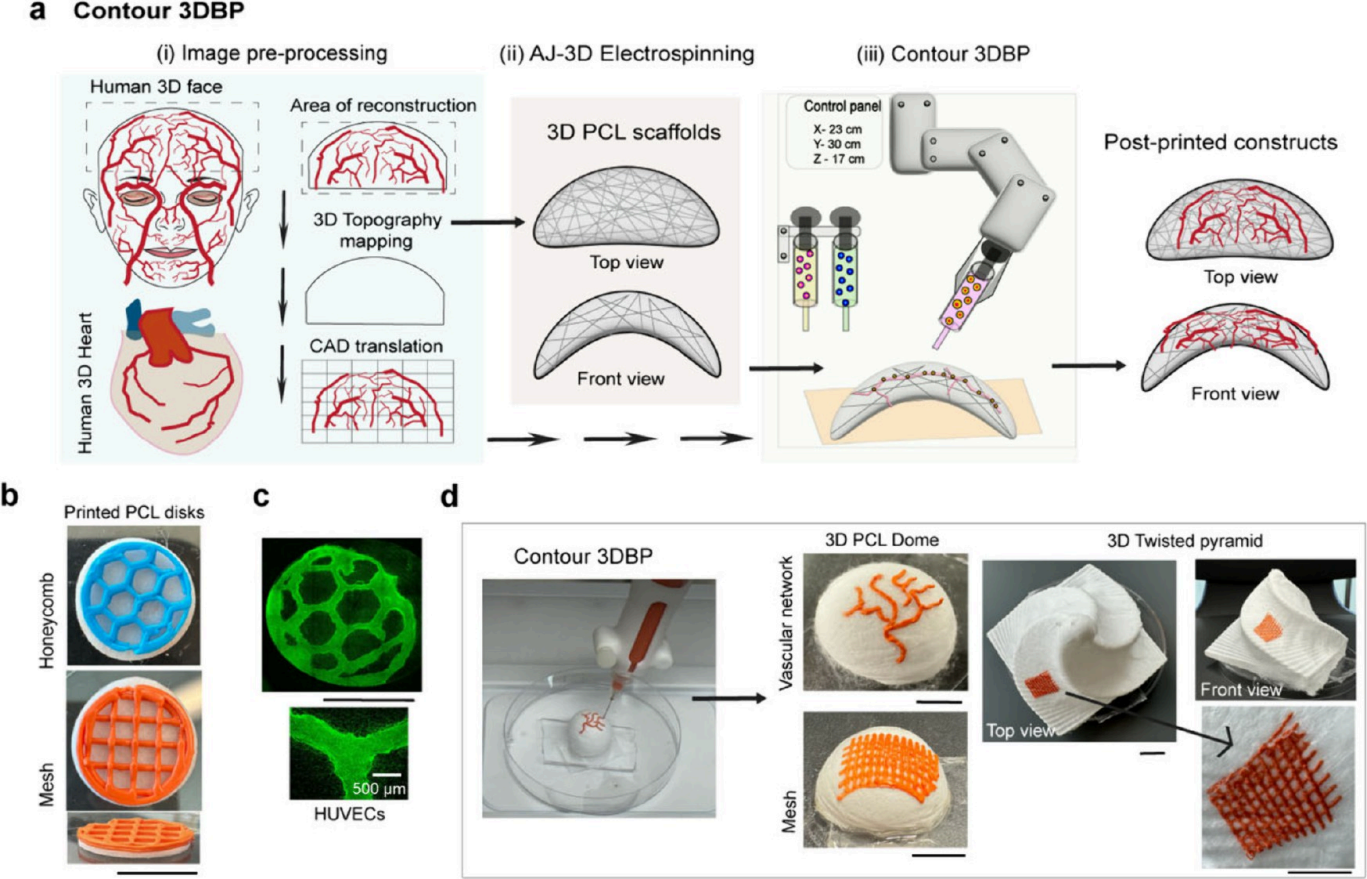
**Contour 3DB printing on geometrically challenging PCL fiber scaffolds**. (a) Schematic representation illustrating the process flow of contour 3DB printing, from image preprocessing, e.g., vascular networks of face and heart, to postprinted constructs. (b) Digital images (left) display honeycomb and mesh patterns printed on PCL fiber disks with colored test inks. (c) Live cell confocal images (right) show HUVECs printed in a honeycomb pattern on a PCL fiber disk after 1 day of in vitro culture. (d) A set of digital images showing the contour printing (left) and contour-printed 3D dome and twisted pyramid PCL fiber constructs with vascular network and mesh patterns (right). Scale bar: 1 cm.

First, a low-viscosity 5% GelMA bioink suspended with HUVECs (1 × 10^6^ cells/mL) was printed onto PCL disks and UV-cross-linked in a honeycomb pattern. By day 3, live cell imaging revealed intact HUVEC honeycomb patterns with high fidelity (Figure 4c). The porous surface of the PCL fiber scaffold absorbed the low-viscosity bioink during printing, while the fiber network minimized ink spreading, enhancing printing fidelity up to 50% when compared with plastic substrate (Figure S5). The use of low-viscosity bioink also reduces cell death caused by shear stress during printing. Hydrogel cross-linking, combined with the fibers, strengthened fiber-hydrogel integration and prevented slipping as supported by peel-off test (Video S5), which is critical for successful printing on 3D surfaces. Another advantage of printing onto these fiber templates is that cells can reassemble by using fiber cues through cell–cell interactions. Test inks were also printed onto 3D fiber templates with high curvature, such as dome and step-pyramid geometries (Figure 4d), creating vascular networks and mesh patterns. These hollow, robust PCL fiber templates retained their structural integrity under dynamic printing conditions, demonstrating their suitability for LbL thickness scaling beyond the fiber plane.

### Volumetric Fabrication via Hydrogel Casting and Freeform Pocket Printing

Leveraging the 3D architecture of PCL fiber scaffolds as hydrogel containment structures enables a transition from surface-level shaping to volumetric fabrication. This approach supports the creation of thicker, anatomically shaped constructs through hydrogel casting and free-form pocket printing within the interconnected spaces of the fiber scaffold. As a proof of concept, a 3D PCL fiber dome scaffold (2.5 cm width × 2 cm height) mimicking the shape of a female breast was fabricated and filled with 1% SA bioink containing mouse embryonic 3T3 fibroblasts. Bioink was deposited LbL using repeated casting and cross-linking cycles (Figure 5a). While this method achieved homogeneous cross-linking, it also introduced interlayer heterogeneity, mimicking the stratification of dermal and epidermal layers (Figure 5b).

**Figure 5:**
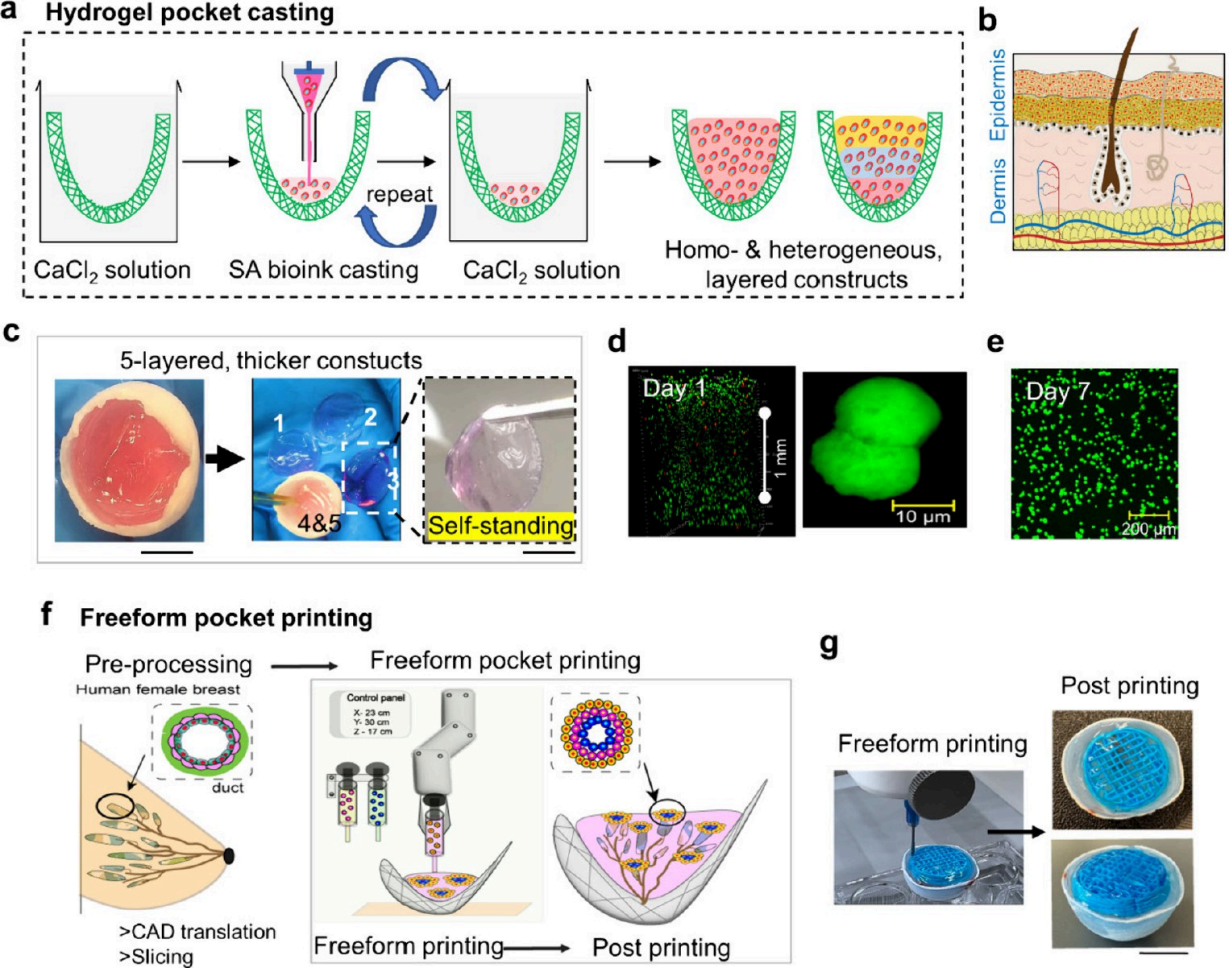
**Volumetric fabrication of anatomical constructs via hydrogel pocket casting and freeform pocket printing.** (a) Schematic of bioink pocket casting for multilayered 3D homogeneous and heterogeneous hydrogel constructs. (b) Representation of tissue heterogeneity across the construct, resembling epidermis and dermis layers. (c) Images showing 1% SA hydrogel cast in a 3D dome-shaped PCL scaffold (*d* = 3 cm), demonstrating layer separation (middle) and self-standing feature (right), confirming uniform cross-linking. (d) Live/dead cell assay showing live (green) and dead (red) cells in a 1.5 mm thick 1% SA hydrogel layer on day 1, with active cell division (right). (e) Live cell images of clustered fibroblasts on day 7. (f) Schematic of pocket freeform printing of thicker constructs, exemplified by the human female breast. (g) Digital images of test ink pocket printing into a 3D PCL dome filled with 0.5% Carbopol support bath (left) and pocket-printed 3D PCL fiber dome (right). Scale bar: 1 cm.

Effective bioink confinement within the plasma-treated PCL fiber dome was ensured by soaking the scaffold in a CaCl_2_ solution. Rapid cross-linking formed a thin hydrogel interface between the fibers and the remaining un-cross-linked bioink, which was subsequently submerged in CaCl_2_ for complete cross-linking. This process yielded five-layered, 2 cm thick constructs (Figure 5c). The PCL fiber dome retained its original shape, and the hybrid constructs exhibited excellent shape memory during repeated squeeze-and-release tests, demonstrating flexibility and robustness (Video S6).

A live/dead assay conducted on the central hydrogel disk on day 1 (Figure 5d) revealed high fibroblast viability (96%) over a measured depth of 1.7 mm with active cell division observed. The cells continued to proliferate over 7 days (Figure 5e), facilitated by 360° medium and oxygen diffusion through the fiber pores. For freeform pocket printing, the fiber-enclosed pocket is first filled with a support bath, followed by direct bioink printing (Figure 5f). A test ink was printed LbL into a PCL fiber dome pocket filled with a Carbopol support bath (Figure 5g and Video S7). After cross-linking and support bath removal, the constructs remain within the fiber pocket, maintaining structural stability and integrity.

### Patterned 2D/3D PCL Fiber Scaffolds for Volumetric Constructs via Modular Integration

Volumetric fabrication can also be achieved through a modular approach by integrating dip-coated or 3DB-printed PCL fiber constructs into the pockets of 3D PCL fiber scaffolds (Figure 6a). This method provides more flexibility in structural arrangements, while the incorporation of fibers enhances cell self-assembly through fiber-guided cues. To promote interlayer cell interactions, we engineered 2D/3D PCL fiber scaffolds with varied density patterns (Figure 6b), significantly reducing PCL content to increase porosity and pore size. This enhances diffusion and supports angiogenic sprouting within the volumetric constructs. Despite the reduced fiber content, the constructs retained mechanical robustness and excellent shape memory.

**Figure 6:**
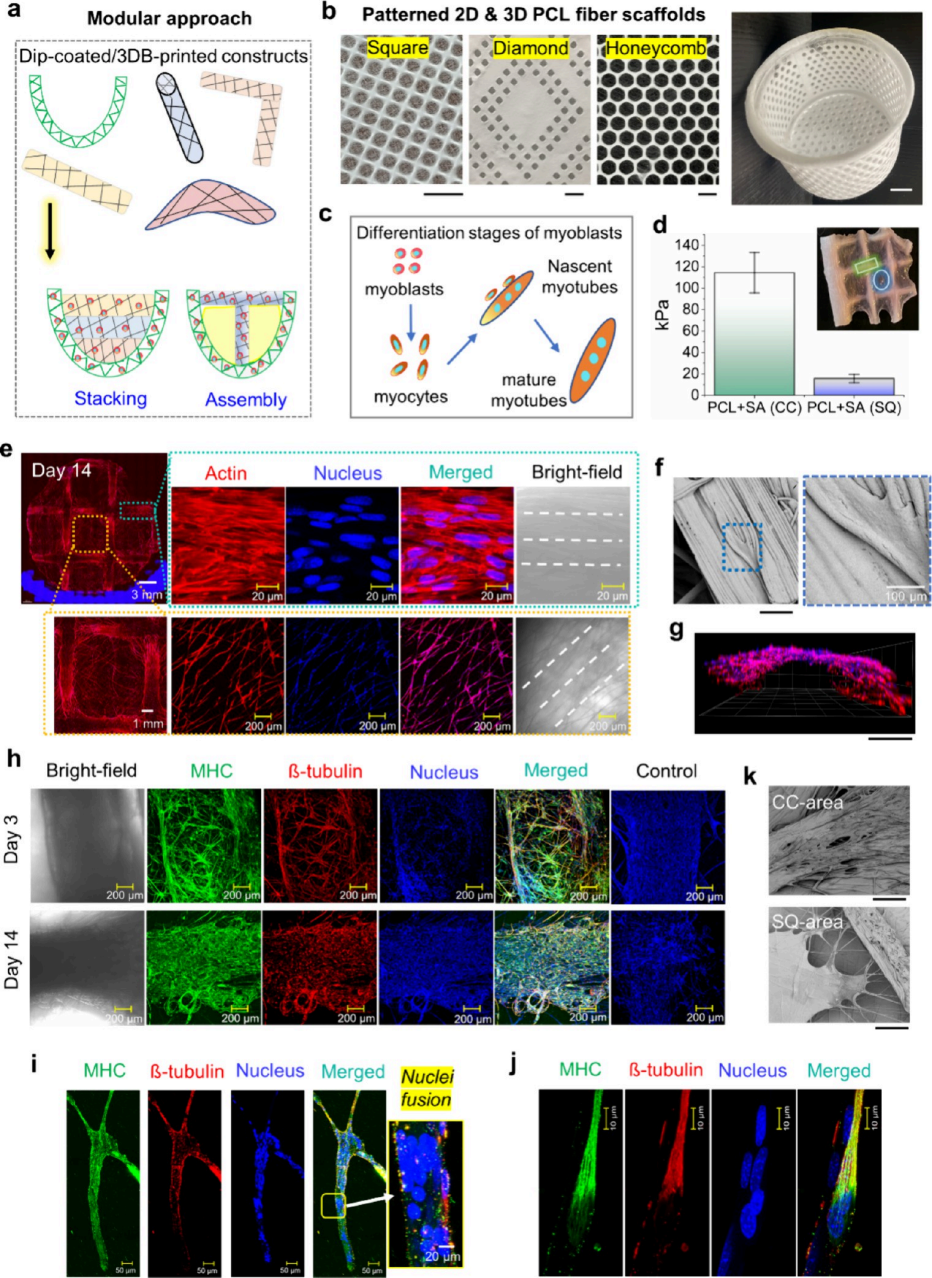
**Modular fabrication of volumetric constructs and myoblast differentiation and alignment in SA dip-coated patterned PCL constructs.** (a) Schematic of thick, layered construct fabrication using a modular approach with stacked hydrogel dip-coated/printed constructs. (b) Digital images of perforated 2D and 3D PCL fiber scaffolds with various patterns made by AJ-3D ES. Scale bar: 1 cm. (c) Schematic of mouse C2C12 myoblast differentiation toward myotube formation. (d) Bar chart comparing Young’s modulus from microindentation of crisscross (CC) and square (SQ) grid-patterned PCL fiber meshes (inset with color-coded area). (e) Confocal images on day 14 showing C2C12 myoblasts on grid fibers (upper) and squares (lower) with actin (red) and nuclei (blue). White dotted lines indicate grid fiber orientation. (f) SEM images showing myoblast morphology on aligned fibers on day 14. (g) 3D-depth profile showing myoblast attachment to the fiber grid. (h–j) Immunohistochemical staining for myosin heavy chain (MHC, green) and beta-tubulin (red) after 3 and 14 days of differentiation. Higher magnification shows (i) myocyte nuclei fusion and (j) parallel alignment of MHC and ß-tubulin along fibers. (k) SEM images on day 28 comparing cell coverage in perforated hybrid scaffolds on crisscross grid and square gap regions. (f, g, k) Scale bar: 300 μm.

A square-patterned construct was dip-coated with a 1% SA bioink containing C2C12 myoblasts (1 × 10^6^ cells/mL) to evaluate corresponding changes in cell density and assembly (Figure 6c). Microstiffness analysis revealed variations in these constructs with lower stiffness inside the squares at 15.6 ± 3.8 kPa and higher stiffness along the fiber grids at 114.1 ± 18.8 kPa, corresponding to fiber density and parallel alignment (Figure 6d). Confocal and SEM imaging after 14 days of culture showed cell density proportional to fiber density, with cells in a parallel arrangement mimicking muscle fibers (Figure 6e,f). Figure 5g also shows these aligned cells taking the curvy shape of the fiber grids. Immunostaining (Figure 6h) confirmed significant differentiation of myoblasts into myocytes by day 3, reaching nearly 100% differentiation by day 14 in the differentiation medium. High-magnification imaging captured active nuclear fusion (Figure 6i) and the parallel alignment of myosin and tubulin along the growth axis (Figure 6j), indicative of multinucleated myotubes. SEM images after 28 days (Figure 6k) revealed excellent cell distribution across both the grids and gaps, demonstrating that the cells eventually bridged larger pores over several hundred micrometers inside the square gaps.

### In Vivo Validation of the AJ-3D ES PCL Fiber Scaffold

To validate the synergistic integration of proposed integrations, a 5 × 5 mm^2^ square PCL fiber sheet with a thickness of 500 μm was implanted subcutaneously between the skin and tendon in the leg of a 7 week old Sprague–Dawley rat (Figure 7a). After 4 weeks of implantation, histological analysis assessed cell compatibility and tissue integration. Hematoxylin and eosin (H&E) staining (Figure 7b) revealed extensive cellular infiltration within the fibrous scaffold, while Masson’s trichrome staining (MTS) showed clear deposition of ECM collagen fibers forming intricate 3D networks (stained blue). These findings confirmed the scaffold’s interconnected, cell-permeable pore structure, effectively bridging the skin and tendon (Figure 7c). This cellular bridging highlights successful proangiogenic migration and the organization of cells into a continuous 3D network, consistent with observations from in vitro studies.

**Figure 7:**
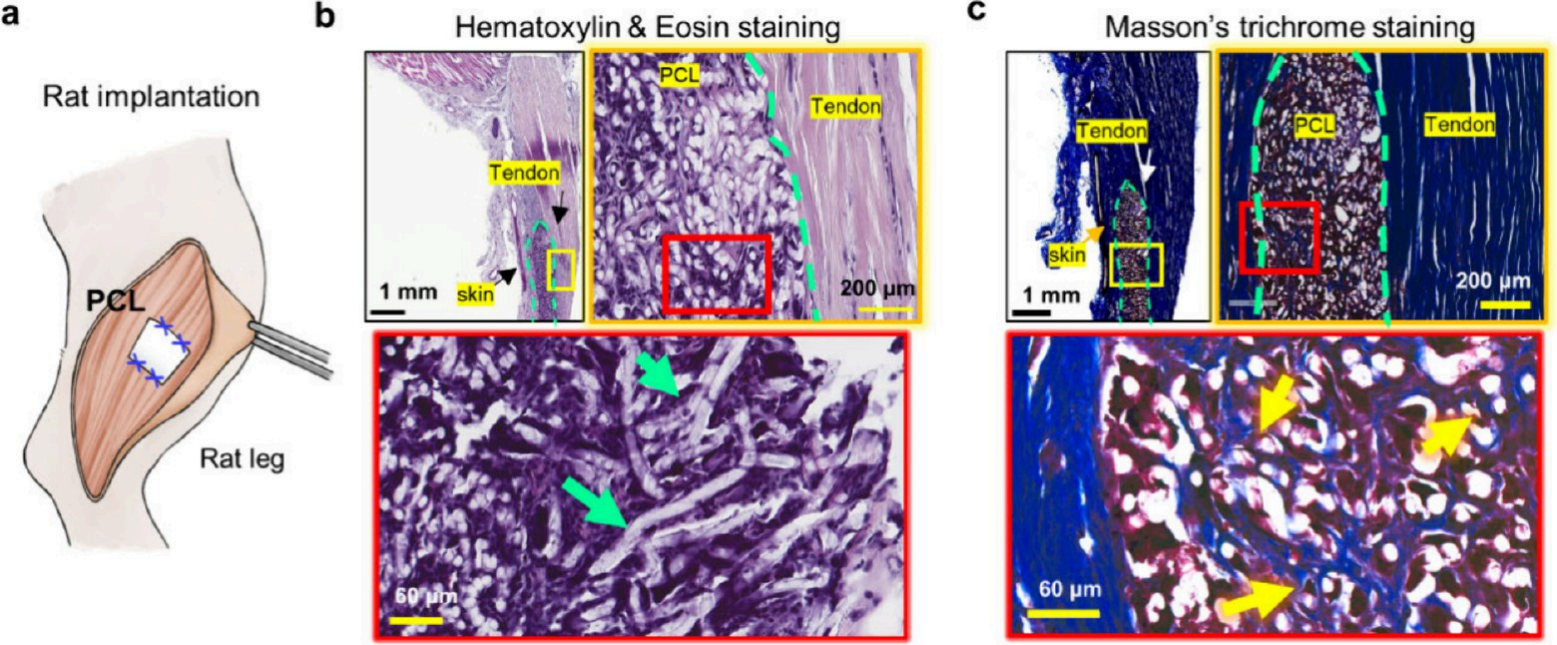
**In vivo functional assessment of AJ-3D electrospun PCL fiber scaffolds in the rat model.** (a) Schematic illustration of subcutaneous surgical implantation of a 0.5 × 0.5 cm^2^ PCL fiber scaffold into the leg of a 7-week-old Sprague–Dawley rat. (b) Hematoxylin and eosin (H&E) staining images (dark purple for nucleus staining, pink for cytoplasm and extracellular matrix (ECM) staining, and white for PCL fiber). (c) Masson’s trichrome staining images (MTS) (blue collagen, dark brown nucleus, pink cytoplasm, and white PCL fiber). Green dotted lines in (b) and (c) delineate the PCL fiber scaffold area between skin and tendon. The yellow and red boxes show corresponding magnified images, with green arrows in (b) indicating PCL fibers and yellow arrows in (c) indicating collagen deposition inside the PCL fiber scaffold.

## Discussion

While 3D bioprinting (3DBP) has shown great promise in disease modeling and drug screening,^49,50^ its clinical translation in regenerative medicine remains in its early stages. A key challenge lies in fabricating clinically relevant constructs that combine scalability, anatomical precision, and functional biomimicry—key factors required to support tissue maturation, vascularization, and long-term viability.^13,51,52^

Building on our previous works that introduced Autopilot jet 3D electrospinning (AJ-3D ES) for fabricating anatomically shaped PCL fiber scaffolds,^35,36^ this study presents a suite of scalable biofabrication strategies that address limitations associated with conventional 3DBprinted hydrogels. Specifically, we demonstrate hydrogel integration approaches, dip-coating, gel casting, and advanced bioprinting techniques such as contour and freeform pocket printing—which expand beyond earlier ES fiber-hydrogel stacking or blending techniques.

Unlike 3DBP, which typically involves slow, bottom-up layer-by-layer (LbL) deposition,^51^ dip-coating provides a rapid and scalable approach to transforming anatomical AJ-3D ES fiber scaffolds into cellularized constructs (Figure 1). This method enables the fabrication of raised, hollow, and anatomically accurate human-scale constructs in a technically simple manner. AJ-3D ES-derived PCL scaffolds also offer superior mechanical resilience and flexibility, distinguishing them from rigid FDM-printed scaffolds or fragile hydrogel-based constructs. For example, SA-dip-coated facial scaffolds retained their form and flexibility while supporting cellularization.

In contrast to conventional electrospun scaffolds that suffer from tightly packed, cell-impermeable pores, AJ-3D ES scaffolds feature an open, gradient porous architecture with pore sizes ranging from 5 to 60 μm—approximately 10 times larger than standard electrospun fibers.^35^ Confocal depth analysis confirmed cell infiltration beyond 200 μm in dip-coated constructs, although deeper imaging was limited by PCL opacity. Notably, SA-coated scaffolds supported the extensive migration and self-organization of MDCK cells and fibroblasts, even without cell adhesion motifs (Figure 2). The fibrous structure, with surface nanopores from solvent evaporation, further enhanced cell attachment and migration. This effect persisted even in 10% GelMA-coated scaffolds with stiffness levels up to ∼50 kPa (Figure 3).

Human tissues exhibit a broad stiffness range—from soft (brain: 0.1–0.5 kPa; liver: 0.5–6 kPa) to stiff tissues (skin: 10–100 kPa; bone: in the GPa range).^53^ Traditional 3D bioprinted hydrogels rely heavily on degradation for tissue maturation, often compromising construct stability.^51^ In contrast, our SA- and GelMA-coated scaffolds, with stiffness between 39 and 250 kPa, supported diverse cellular outcomes, including epithelial sheet formation, fibroblast clustering, and co-cultured liver models. The structural stability of the PCL scaffold over 28 days underscores its potential to support long-term tissue maturation and integration. Enhanced confluency observed in the liver co-culture model further demonstrates how low-viscosity, ECM-based, high-cell-density bioinks in dip-coated constructs accelerate tissue formation. Such constructs show strong promise for applications in wound healing and reconstructive surgery by guiding self-organization through intrinsic cellular sorting or host biochemical cues (Figure 8a).

**Figure 8:**
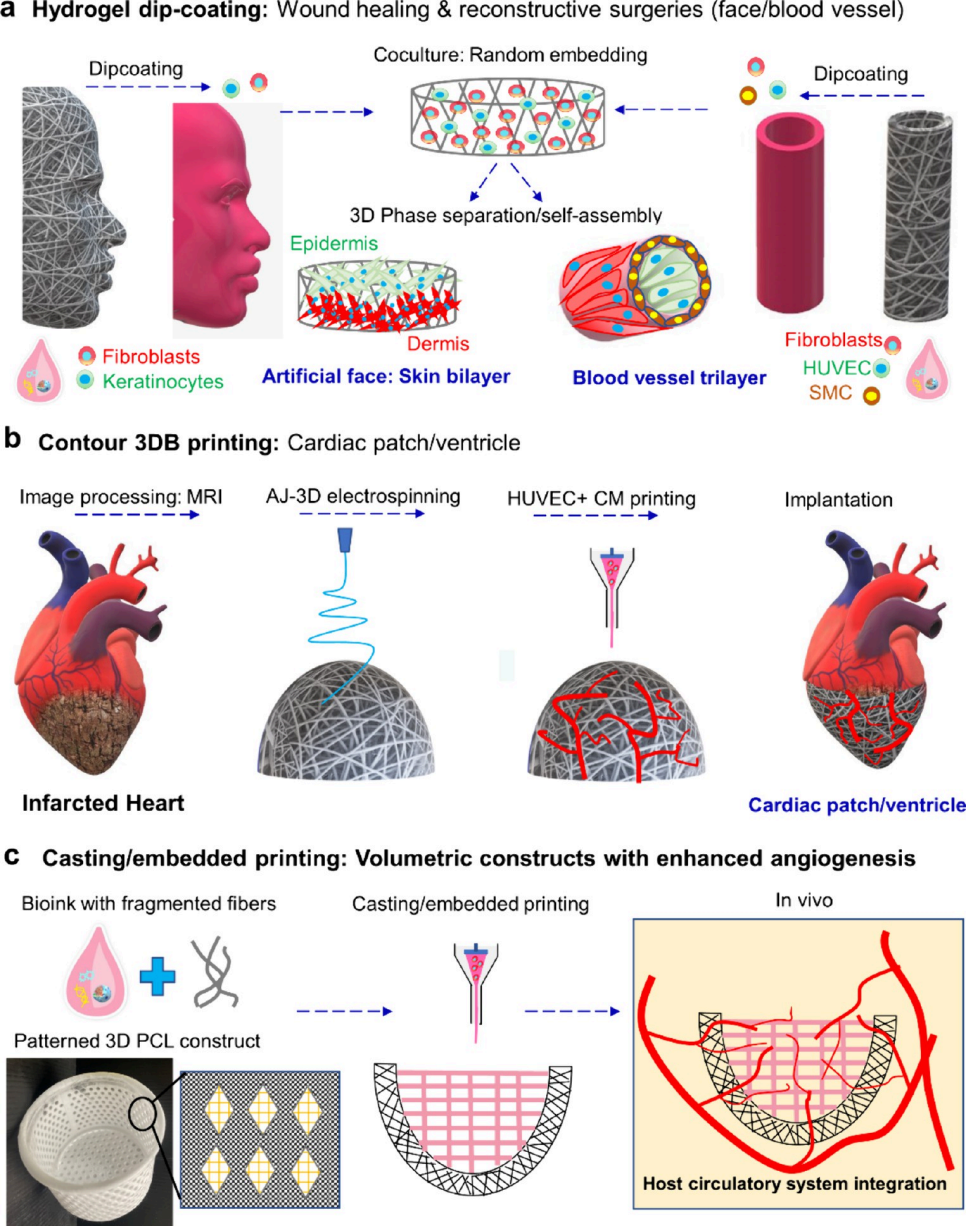
**Mechanistic illustration summarizing patient- and organ-specific topographic and volumetric construct biofabrication for diverse TE applications.** (a) Anatomical PCL fiber scaffolds mimicking facial and tubular (blood vessel) topographies are infused with tissue-specific cells—fibroblasts and keratinocytes for skin TE and fibroblasts, HUVECs, and smooth muscle cells (SMCs) for engineered blood vessels. Following incubation, constructs are implanted, where cell self-assembly and organization are guided by scaffold architecture and host biochemical cues. (b) Biofabrication of a cardiac patch/ventricle for myocardial infarct repair using in situ contour 3DB printing. HUVEC- and cardiomyocyte (CM)-laden bioinks are separately prepared with HUVECs printed into vascular networks on PCL fiber scaffolds. After incubation, the construct is implanted to promote functional myocardial restoration. (c) For the fabrication of ideal volumetric constructs, bioinks blended with fragmented fibers and patterned PCL fiber scaffolds are proposed to enhance cellular function in printed constructs and promote angiogenesis for improved integration with the host circulatory system.

Although in situ 3DBP has been explored for direct surgical applications, challenges remain in printing complex geometries at wound sites, maintaining hydrogel integrity in physiological environments, and ensuring integration.^54,55^ Our novel contour 3DB printing approach circumvents these challenges by utilizing robust AJ-3D ES fiber scaffolds, which enhance print fidelity for low-viscosity (5% GelMA) bioinks (Figure 4 and Figure S5). This setup promotes high cell viability and retention, indicating potential use in cardiac patches and ventricular grafts for myocardial regeneration (Figure 8b). In contrast, conventional hydrogel-based delivery systems often suffer from poor cell retention, leading to limited engraftment and therapeutic efficacy.^56,57^

In addition to dip-coating and contour 3DB printing, we explored volumetric construct fabrication through gel casting, freeform pocket printing, and modular assembly (Figures 5 and 6). In these strategies, AJ-3D ES fiber scaffolds provided both mechanical support and spatial confinement, allowing hydrogels to shift from a structural to a primarily biological role. This enabled the use of minimally cross-linked, high-cell-density bioinks.^56,57^ In gel-casted constructs, fibroblast viability exceeded 96%, highlighting that the scaffold’s porous architecture facilitates nutrient and oxygen diffusion into hydrogel. Earlier studies show that limited and nonuniform porosity of casted gels imposes diffusion limits in gels with a thickness beyond 2 mm, which is influenced by hydrogel stiffness and shape.^58,59^

To overcome these diffusion limits, freeform pocket printing within a Carbopol support bath enabled pattern-controlled deposition, offering improved scalability and spatial resolution. The AJ-3D ES platform allows high freedom in fiber arrangement, including patterned geometries such as criss-cross-aligned grids, which facilitate myocyte alignment, mimicking striated muscle bundles. This guided cellular organization, coupled with larger pores for improved diffusion and angiogenesis, enhances the overall construct functionality. Additionally, incorporating fragmented fibers into bioinks provided fiber-mediated guidance cues^32^ to promote structured cell assembly (Figure 8c).

In vivo validation in a rat model showed extensive cell infiltration and robust ECM (collagen) deposition within implanted scaffolds placed between the tendon and skin (Figure 7). These findings confirm that the porous AJ-3D ES scaffolds synergize effectively with hydrogels across different strategies, supporting deep cell penetration, uniform distribution, and long-term viability—essential parameters for functional tissue regeneration.

Regarding size scalability, FDM-based supports can fabricate organ-scale structures but lack cellular fidelity, while freeform printing of cellular constructs is generally restricted to lab-scale formats.^18–20^ In contrast, AJ-3D ES-based integration strategies offer topographic and volumetric scalability, with the AJ-3D ES platform previously shown to support organ-scale scaffold fabrication.^36^ Thus, both anatomical shaping and volumetric assembly on clinically relevant scales are achievable. The choice and suitability of these integration strategies may be tailored based on the specific requirements and characteristics of the target tissue type.

## Conclusions

This study demonstrates the transformative potential of the AJ-3D ES technique in overcoming the scalability-functionality trade-off inherent to 3DB printing techniques through seamless and flexible integration with hydrogels, establishing a versatile platform for diverse tissue engineering. Future advancements optimizing fiber architecture and bioink properties enable tissue/organ-specific biomimetic scaffold development, bridging the gap between engineered constructs and native tissue complexity. Anatomical shaping of AJ-3D ES scaffolds holds promise in organoid engineering for regenerative medicine, where fiber integration potentially enhances culture stability and promotes shape-guided expansion.

## Experimental Section

### Materials

Polycaprolactone (PCL, 80,000 mW), gelatin powder derived from porcine skin (Type A, SLCC7838), methacrylic anhydride (MA), 2-hydroxy-4′-(2-hydroxyethoxy)-2-methylpropiophenone (Irgacure 2959), chloroform, diethyl ether, sodium alginate (SA), calcium chloride (CaCl_2_), and phosphate buffer solution (PBS) were purchased from Sigma-Aldrich. The Carbopol ETD 2020 polymer was obtained from Lubrizol (Wickliffe, USA).

### Electrospinning 2D/3D PCL Fiber Scaffolds

The preparation of the PCL solution and the electrospinning process were performed based on our prior study. Briefly, PCL beads were dissolved in a chloroform and diethyl ether (9:1) solvent system to achieve a 15.78% (w/v) PCL solution, which was stirred magnetically for 4 h to ensure homogeneity. A 22G blunt needle was attached to a 20 mL syringe containing the PCL solution, which was then loaded into a syringe pump. The dispensing rate was set to 3 mL/h, and a high voltage of 10 kV was applied to the needle using a high voltage power supply. Metal templates were connected to the ground terminal to establish the electric field and were positioned 10 cm from the needle tip. During the fiber deposition process, a single jet emerging from the needle tip undergoes rapid self-jet switching between two modes, namely, armed jet and whipping mode, back and forth indefinitely, that expands the fiber deposition area and thickness alternatively over time by following the electric field profile across the collector without the need for the manipulation of the collector/stage. This automated fiber deposition process is referred to as “autopilot jet” in this context.^35,36^ After electrospinning, the PCL fiber scaffolds were removed from the collectors and stored in a desiccator.

### Cell Culture

The following cell lines were used for in vitro experiments: MDCK epithelial cells, MDCK epithelial cells transfected with GFP and RFP expressing tight junction protein ZO-1 and H2A histone, mouse embryonic 3T3 fibroblasts, human umbilical vein endothelial cells (HUVECs), HepG2 cells, and mouse C2C12 myoblasts. Complete culture medium for MDCK, 3T3 fibroblasts, HepG2, and C2C12 cells consisted of DMEM supplemented with 10% fetal bovine serum (FBS) and 1% penicillin-streptomycin (PS). For C2C12 differentiation, 10% FBS was replaced with 2% horse bovine serum (HBS). HUVECs were cultured in a Vasculife VEGF Endothelial Medium Complete Kit (LifeLine Cell Technology). Frozen cell vials stored in liquid nitrogen were thawed, cultured in medium, and incubated at 37 °C with 5% CO_2_. Media were replenished every other day until 80% confluence was reached, after which cells were trypsinized, centrifuged, and subcultured. A hemocytometer was used for cell counting.

### SA and CaCl_2_ Solution Preparation

A 1% SA solution was prepared in complete DMEM for MDCK cells and 3T3 fibroblasts, stirred for 2 h at 37 °C, and degassed in a desiccator for 30 min before cell addition to create bioink. The SA cross-linking solution of 80 mM CaCl_2_ was prepared in autoclaved Milli-Q water and sterile-filtered using a 0.22 μm PVDF syringe filter.

### GelMA Synthesis and Solution Preparation

Highly substituted GelMA was synthesized following our previously reported protocol with slight modifications.^25^ Briefly, 5 g of gelatin from porcine skin (gel strength 300, Sigma) was dissolved in PBS at 50 °C to create a 10% (w/v) solution. Methacrylic anhydride (4 mL) was added drop by drop to the reaction chamber, and stirring was continued for 3 h at 50 °C. The reaction was halted by adding excess warm PBS and stirring for 15 min. The GelMA solution was dialyzed against DI water using a dialysis membrane with a 10 kDa molecular weight cutoff (Thermo Scientific SnakeSkin Dialysis Tubing) for 7 days with 2–3 times daily water change to remove unreacated methacrylic anhydride. The dialyzed solution was lyophilized for 7 days and stored at −20 °C (protected from light). GelMA was reconstituted in PBS with 0.5% (w/v) Irgacure 2959 to create 5 and 10% (w/v) solutions by immersing in PBS and incubating at 37 °C for 5–10 h. The cross-linking mechanism of GelMA was drawn using ChemDraw.

### SA and GelMA Bioink Preparation

In this study, a total of seven bioinks were prepared with different cell types and concentrations. The first bioink consisted of 1% (w/v) SA with MDCK cells at a concentration of 1 × 10^6^ cells/mL. Similarly, 1% (w/v) SA was also prepared with MDCK cells transfected with GFP and RFP at 1 × 10^7^ cells/mL, as well as with mouse embryonic 3T3 fibroblasts at the same cell concentration. For the GelMA-based bioinks, 5% (w/v) GelMA was prepared with HUVECs at a concentration of 1 × 10^6^ cells/mL for contour 3DB printing, 5% (w/v) GelMA was prepared with HUVECs and HepG2s at concentrations of 2 × 10^6^ and 10 × 10^6^ cells/mL, respectively, for co-cultured experiment, while 10% (w/v) GelMA included HUVECs at a concentration of 2 × 10^6^ cells/mL. Lastly, 1% (w/v) SA was mixed with mouse C2C12 myoblasts at 1 × 10^6^ cells/mL. All bioinks were prepared using their respective cell culture media, with the co-culture medium for the 5% (w/v) GelMA bioink containing HUVECs and HepG2s prepared in a 20:80 ratio.

### Plasma Treatment of PCL Fiber Scaffolds

Prior to dip-coating and pocket casting, all hydrophobic 2D and 3D ES PCL fiber scaffolds were subjected to oxygen plasma treatment using a Harris plasma treater (USA) at high power, 500 mTorr, for 1 min to introduce polar groups, enhancing wettability. The treated scaffolds were then UV-sterilized for 1 h on each side and washed with 70% alcohol and DI water (3 times, 15 min intervals each).

### SA and GelMA Dip-Coated Construct Preparation

Circular PCL fiber disks (15 mm diameter) were used for dip-coating experiments except for patterned disks in muscle tissue engineering with C2C12 myoblasts. In the SA-based dipcoating process, PCL fiber disks were dipped into the SA bioink for 30 s, followed by immersion in 80 mM CaCl_2_ solution for 1 min. SA dipcoated disks were rinsed in DI water for 15 s to remove excess CaCl_2_ before incubation. The same process was followed for the for human face-mimicking 3D PCL scaffold, but SA solution devoid of cells was used. For the GelMA-based process, disks dipped in 5% (w/v) and 10% (w/v) bioinks for 30 s were UV-cross-linked (Omicure S2000 UV curing system, USA) for 90 s (60 s for the top side, 30 s for the bottom), with UV intensity set at 10 and 20 mW/cm^2^ for low and high cross-linking, respectively.

### Tensile Strength Analysis

To study the influence of hydrogel integration on the bulk mechanical properties of dip-coated constructs, tensile strength analysis (ComeTech, Taiwan) was performed on the following samples: PCL alone, 1% SA, 5% and 10% GelMA dip-coated constructs. The samples were precut into rectangular pieces 4 × 1 cm^2^ using a laser cutter, and the power and speed were 18% and 20 mm/s. The rectangular PCL strips were then dipped with respective bioinks and cross-linked accordingly. These samples were placed between the grips, and the mesh was stretched at a speed of 50 mm/min. Strain–stress values and Young’s modulus were automatically calculated using Instron software.

### GelMA 3DB Printing

Circular PCL fiber disks (diameter = 15 mm) were printed with mesh and honeycomb patterns for the modular approach, where postprinted disks were integrated into the dome pocket to facilitate modular assembly of the structures. The cartridge containing 5% (w/v) GelMA bioink with 1 × 10^7^ HUVECs per mL, maintained at room temperature, was used to print a honeycomb pattern onto the PCL disk using the same parameters given in the freeform pocket printing. The construct was then incubated in VascuLife VEGF Endothelial Medium (Lifeline Cell Technology, Maryland, USA) up to 3 days and was then fixed in 4% formaldehyde solution and imaged using a Stellaris 8 confocal microscope (Leica, Wetzlar, Germany).

### Contour 3DB Printing

Contour 3DB printing was carried out on 3D PCL fiber scaffolds with dome-shaped and twisted pyramid structures using the in situ 3DB printer BioAssemblyBot 500 (Advanced Solutions, USA). The surface of the replicas was initially scanned using a si*x*-axis robotic arm with a 3D Laser Scan BioAssemblyTool. Subsequently, patterns, including mesh and vascular networks, were drawn on the scanned surfaces by using the native TSIM software (Advanced Solutions). These files were uploaded back into the machine, and the patterns were translated by printing onto the 3D ES PCL fiber scaffolds along the contours using a six-axis robotic arm equipped with a printing head. The printing parameters were as follows: nozzle, 22G; printing speed, 10 mm/min; and printing pressure, 150 kPa.

### 3DB Printing Fidelity and Hydrogel Peel-Off Study

5% GelMA bioink was printed onto an AJ-3D ES PCL fiber sheet into circular (*d* = 2 cm) with square grids, followed by UV cross-linking for 30 s. Bioink was printed into two layers horizontally and vertically to form square grids with 0.5 mm line width, and the total thickness was measured to be 1 mm (from the fiber plane). The hydrogel printed fiber sheet then was subjected to bending both inward and outward. To test the GelMA hydrogel integrity within the fiber substrate, a tweezer was used to peel off the hydrogel, which showed firm attachment to the fiber, confirming our claim.

### Hydrogel Pocket Casting into a 3D PCL Fiber Dome

In this study, 3T3-fibroblast-suspended 1% (w/v) SA bioink was prepared by using pocket casting. Plasma-treated 3D PCL fiber domes served as the substrates for both experiments. For SA pocket casting, 1% (w/v) SA bioink was cast into the pocket of plasma-treated 3D PCL fiber domes. The domes were dipped in CaCl_2_ solution to wet them, after which 1 mL of bioink was pipetted into the pocket. They were then reimmersed in the 80 mM CaCl_2_ cross-linking solution for 1 min. This cycle was repeated five more times to completely fill the pockets with the hydrogel. The hydrogel-casted 3D domes were then immersed in the cultured medium. The medium was changed every other day until live cell imaging.

### Carbopol Support Bath Preparation for Freeform Pocket Printing

The Carbopol support bath for freeform pocket printing was prepared by dissolving Carbopol (ETD 2020, Lubrizol) in DI water at a concentration of 0.5% (w/v) under magnetic stirring at room temperature overnight. Next, the pH was adjusted to 7–7.5 using 4 M NaOH until the suspension became clear and highly viscous. The Carbopol was then centrifuged at 2000*g* for 10 min for degassing and stored at 4 °C.

### Freeform Pocket Printing into a 3D PCL Fiber Dome

For freeform pocket printing, the extrusion-based bioprinter BIO X (Cellink, Sweden) was used. The 3D image of the dome-shaped 3D PCL fiber scaffold was scanned by using the BioAssemblyBot (BAB) 500 3D Laser Scan BioAssemblyTool, and in-fill patterns were created by using Autodesk Fusion 360. The stl files were uploaded to the BioX and sliced using the internal slicer for printing within the Carbopol-filled 3D dome-shaped PCL fiber pocket. Red and blue test inks were prepared by mixing Nivea hand cream with food colors, and printing was conducted using test inks with the following optimized parameters: nozzle 22G, printing speed of 10 mm/min, and printing pressure of 35 kPa.

### Cultured Samples Fixing Time Points

To assess cell proliferation, morphology, and assembly in the hybrid constructs, fluorescent and immunohistochemical staining were performed. The hybrid constructs were fixed with 4% formaldehyde in PBS for 1 h after three PBS washes (except those subjected to live cell imaging) at predetermined time points: MDCK cells on days 5, 14, 21, 35; GFP and RFP transfected MDCK cells (live cell imaging) on day 14; 3T3 fibroblasts on days 7, 14, 21, 28; HUVECs and HepG2s on days 3, 14, 21; HUVECs on days 14, 28; C2C12 on days 7, 14, 17 (day 3: differentiation), 28 (day 3: differentiation); (pocket casted) 3T3 fibroblasts (live cell imaging) on days 1, 7; and contour 3DB-printed HUVEC on day 3.

### Fluorescent Staining

Fluorescent staining of actin and nuclei was carried out using Alexa Fluor 488 Phalloidin and DAPI (Thermo Fisher Scientific) according to the manufacturer’s protocols. Briefly, fixed constructs were first washed with PBS, followed by incubation with 0.1% Triton-X in PBS for 5 min, soaking in 1% BSA for 1 h, and then adding Phalloidin to a final concentration of 20 μm for 30 min. A 300 nM DAPI stain solution was applied, covering the construct and incubating for 5 min. PBS washes were performed after each staining step. Samples were mounted on a cover glass using ProLong Gold Antifade Mountant (ThermoFisher) and imaged using an Inverted Confocal plus Super Resolution Microscope (LSM 780 + ELYRA), Zeiss.

### Immunohistochemical Staining

Constructs designated for immunohistochemical staining were incubated with primary and secondary antibodies following the Triton-X 100 and BSA incubation steps. The antibodies used were as follows: MDCK cell epithelial cadherin protein expression: recombinant Anti-E Cadherin primary antibody (1:500 dilution) (ab308347; Abcam)/Goat Anti-Rabbit IgG H&L (Alexa Fluor 594) secondary antibody (1:500 dilution) (ab150080; Abcam); C2C12 myoblast differentiation: Anti-myosin heavy chain mouse monoclonal primary antibody (4:1000 dilution) (ab37484; Abcam)/Goat Anti-Mouse IgG H&L (Alexa Fluor 488) secondary antibody (1:1000 dilution) (ab150113; Abcam) and Antibeta Tubulin rabbit monoclonal primary antibody (1:200 dilution) (ab179513; Abcam)/Goat anti-rabbit IgG H&L secondary antibody (Alexa Fluor 594) (1:500 dilution) (ab150080; Abcam); and for HUVECs (GFP positive): Anti-GFP secondary antibody (Alexa Fluor 488) (ab13970; Abcam), HepG2: Anti-HNF4A monoclonal antibody (1:200 dilution)/Goat anti-mouse H&L secondary antibody (Alexa Fluor 594) (1:500 dilution) (ab150080; Abcam). Primary antibodies were incubated overnight at 4 °C, followed by secondary antibody incubation for 1 h the next day. Samples were mounted on a cover glass using mounting media and imaged using an Inverted Confocal plus Super Resolution Microscope (LSM 780 + ELYRA), Zeiss, and Leica SP8 Inverted Microscope. Image analysis was performed using ImageJ/FIJI, National Institute of Health (NIH), USA.

### Live/Dead Cell Viability Assay

The live/dead cell viability assay (ab287858; Abcam) was conducted on the pocket-casted 3T3 fibroblast-embedded SA hydrogel on day 1 following the manufacturer’s protocol. A solution consisting of 2 μL of Cell Dye II/Live Cell Staining Dye and 1 μL of Dead Cell Staining Dye was mixed in 1 mL of the Assay Buffer XXVII/Assay Buffer. This solution was added to the cast SA hydrogel, which had been washed with PBS three times and observed immediately under the Zeiss inverted microscope (LSM 780 + ELYRA).

### Live/Dead Assay Cell Viability Calculation

Confocal images from the live/dead assay were analyzed using ImageJ/FIJI, NIH, USA, with cell viability calculated according to Allevi protocols. Briefly, images were first Z-stacked and then Z-projected to merge all slices into a single image. The green and red channel signals were split, converted to 8-bit, and adjusted to grayscale images with optimized thresholds. Cell counts were generated using the “Analyze Particles” option, and cell viability was calculated using the formula

$$Cell\;viability\;\left(\%\right)=\frac{green}{\mathrm{total cells}\;\left(\mathrm{green}+\mathrm{red}\right)\times 100\%}$$

### SEM Imaging

SEM image analysis of fiber morphology (Plain PCL fiber disk) and cell morphology and ECM coverage on C2C12 dip-coated SA hybrid constructs were conducted after 28 days of incubation in regular and differentiation media (14 days each). Formaldehyde-fixed scaffolds were treated with sequential ethanol concentrations (30%, 50%, 75%, 90%, and 100%) at 15 min intervals. Hexamethyl disilazane (HMDS) was added to the constructs, which were left in a laminar hood overnight before SEM imaging. A 5 nm thick gold film was sputter-coated onto the samples for 1 min, and imaging was performed using a JEOL SEM at 5 kV.

### Microindentation Analysis

The stiffness of the dip-coated hybrid constructs (*n* = 4 per group) was evaluated using a microindentation test, following our previously established protocol with a Mach-1 micromechanical system (Biomomentum Inc., Quebec, Canada). A 500 μm spherical indenter tip was employed to indent the constructs from the outer surface to a depth of 100 μm at a controlled rate of 2 μm/s. Stiffness (*S*) was determined by analyzing the slope of the linear portion (initial 5–20%) of the force–displacement unloading curves for each construct. Subsequently, the reduced elastic modulus (*E*_r_) was calculated following the equation^25,60^

$$E_{r}=\frac{\sqrt{\pi }}{2\beta }\frac{s}{\sqrt{A\left(h_{c}\right)}}$$

where *A*(*h*_c_) represents the projected contact area at the indentation depth *h*_c_ and β = 1 is a dimensionless constant derived from the following equation

$$A\left(h_{c}\right)=2\pi Rh_{c}-\pi {h_{c}}^{2}$$

in this context

$$h_{c}=h_{\max}-\varepsilon \frac{P_{\max}}{S}$$

where *P*_max_ and *h*_max_ denote the maximum unloading force and displacement, respectively. The dimensionless constant of ε = 0.75 was used for the spherical indenter in this study.

The Young’s modulus (*E*) was subsequently determined using the following equation

$$\frac{1}{E_{r}}=\frac{1-v^{2}}{E}+\frac{1-{v_{i}}^{2}}{E_{i}}$$

where *E*_i_ is the elastic modulus of the indenter tip (= 2 GPa) and *v* and *v*_i_ are the Poisson’s ratio of the hydrogel (= 0.5) and the indenter tip material used (= 0.5), respectively.

The calculated values were then plotted in bar graphs using Origin Pro 2018 software.

### In Vivo Experiments and Histological Analysis of Cryo-Sectioned Tissue Samples

The animal model protocol was approved by the National Laboratory Animal Center, National Applied Research Laboratories (NARL) (Taipei, Taiwan; NLAC-111-M-027). A square PCL fiber sheet (0.5 cm × 0.5 cm) with a thickness of 500 μm was surgically implanted subcutaneously into the leg of a 7-week-old Sprague–Dawley rat. The in vivo study was conducted at the National Laboratory Animal Center in Taipei. Four weeks postimplantation, the rat was euthanized to assess cellular arrangement within the scaffold. Hematoxylin and eosin (H&E) and Masson’s trichrome stains of cryo-sectioned tissue samples were performed with the support of the Taiwan Mouse Clinic, Academia Sinica, and Taiwan Animal Consortium.

## Supplementary Material

















## Notes

Supplementary figures showing a SEM image of fused buckled fibers, a schematic representation of the SA dip-coating process, and confocal microscopy images of 3T3 fibroblasts (PDF)
Video S1: Flexibility and shape memory of the 3D human face anatomical PCL fiber scaffold by the AJ-3D electrospinning technique (MP4)
Video S2: SA-dipcoated face construct demonstrating pliability and resiliency, mimicking native skin characteristics (MP4)
Video S3: 3D depth profile analysis video showing the cell morphology and distribution across the SA-dipcoated hybrid construct after 5 days into culture (MP4)
Video S4: Monolayer of MDCK and lumenogenesis in the SA dipcoated hybrid construct after 35 days into culture (MP4)
Video S5: GelMA hydrogel stability testing via bending and peel-off postprinting onto the AJ-3D ES fiber sheet (MP4)
Video S6: Structural integrity and shape memory of pocket casted SA hydrogel (MP4)
Video S7: Freeform pocket 3DB printing into the Carbopol-filled dome-shaped 3D PCL pocket using a cell ink 3D bioprinter (MP4)
