# Supplementary material for: Scalable Biofabrication of Functional 3D Scaffolds via Synergy of Autopilot Single-Jet Electrospun 3D PCL Fiber Scaffolds and Cell-Laden Hydrogels
Source: ACS Appl Mater Interfaces. 2025 Jul 22;17(34):47878–93. doi: 10.1021/acsami.5c07425 (PMC12400274; doi:10.1021/acsami.5c07425)
Supplement: Supplementary file 1 [file am5c07425_si_001.pdf]

Supporting information

Scalable Biofabrication of Functional 3D Scaffolds via Synergy of Autopilot Single-Jet Electrospun 3D PCL Fiber Scaffolds and Cell-laden Hydrogels

Balchandar Navaneethan, Mehdi Salar Amoli, Yen-Ching Yang, Sarah Rezapourdamanab, Chiao-Yu Tseng, Yamini Singh, Chin-Lin Guo, Vahid Serpooshan, Chia-Fu Chou*

[1]Institute of Physics, Academia Sinica, Taipei 11529, Taiwan, ROC

[2]Biomedical Translational Research Center, National Biotechnology Research Park, Academia Sinica, Taipei 11571, Taiwan, ROC

[3]Wallace H. Coulter Department of Biomedical Engineering, Emory University School of Medicine and Georgia Institute of Technology, Atlanta, GA 30322, USA

[4]Department of Pediatrics, Emory University School of Medicine, Atlanta, GA 30322, USA

[5]Children's Healthcare of Atlanta, Atlanta, GA 30322, USA

[6]Research Center for Applied Sciences, Academia Sinica, Taipei 11529, Taiwan, ROC

***Corresponding author:** cfchou@phys.sinica.edu.tw

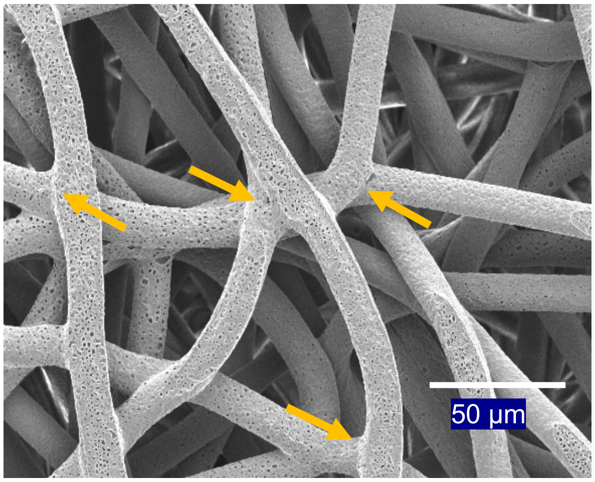

Figure S1. SEM image shows PCL fiber morphology, with yellow arrows pointing fiber fusion at junctions.

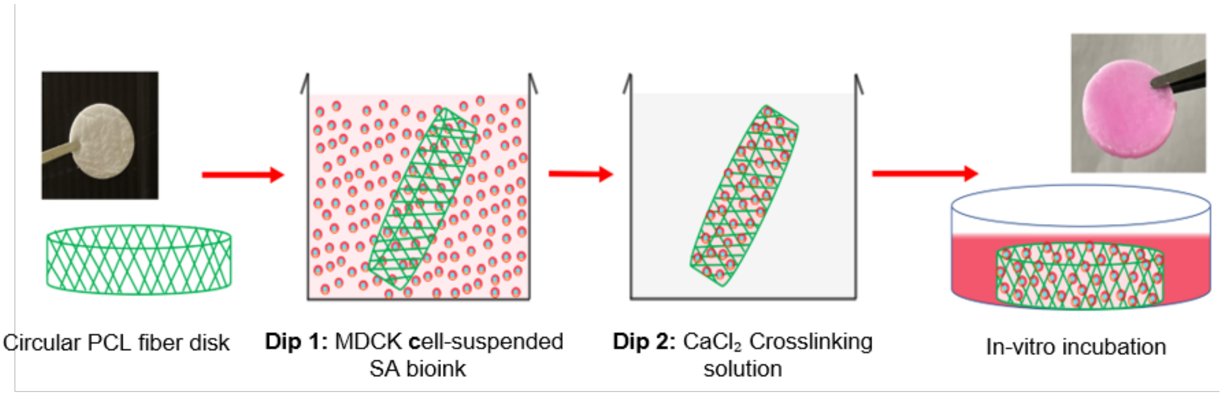

Figure S2. Schematic representation of the two-step dip-coating process of SA hydrogel. PCL scaffolds are first coated with MDCK cell-suspended SA bioink and then immersed in a $CaCl_2$ crosslinking solution before in vitro incubation.

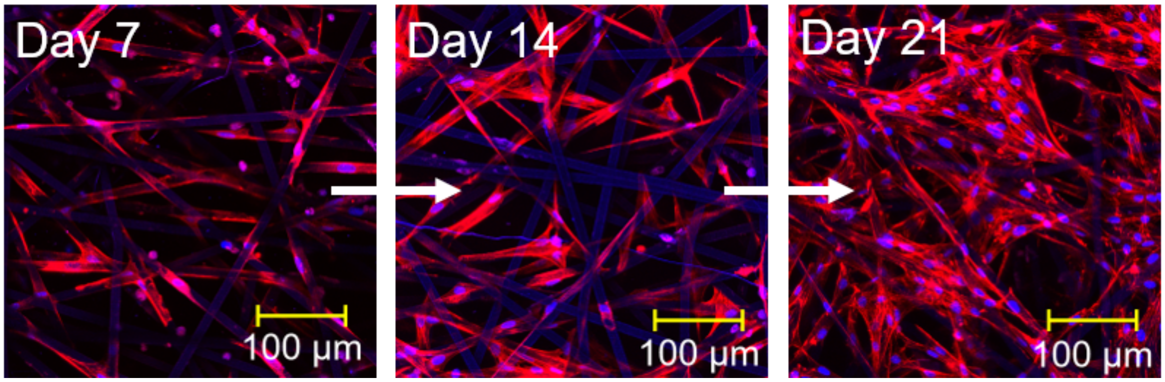

Figure S3. Confocal microscopy Z-stack maximum intensity projection images showing embedded 3T3 fibroblasts in SA dip-coated constructs over 21 days.

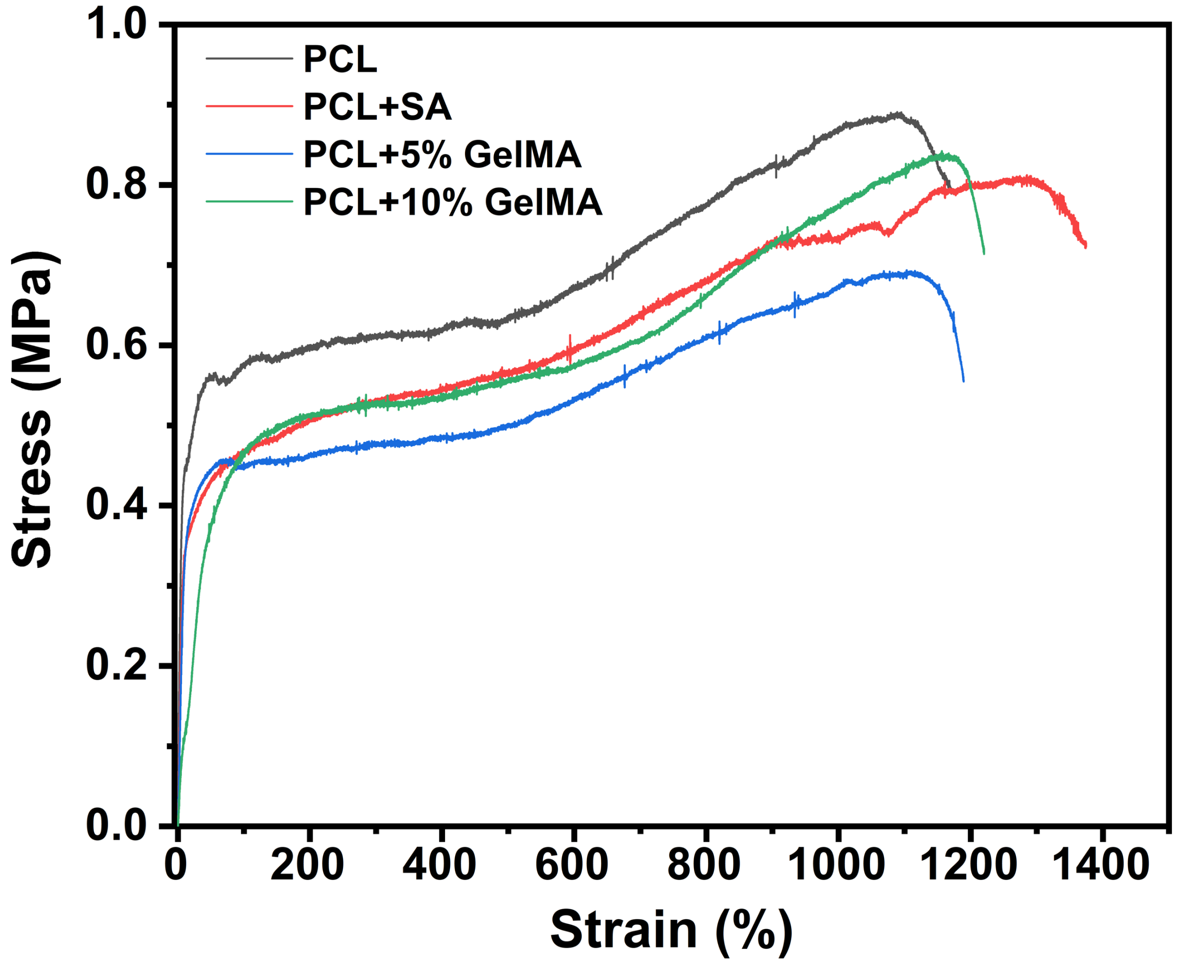

Figure S4. Tensile strength analysis of ES PCL fiber scaffolds and dip-coated constructs. Strain Vs. stress graph comparing the tensile strength of PCL, PCL+SA, PCL+5% GelMA, and PCL+10% GelMA dip-coated constructs.

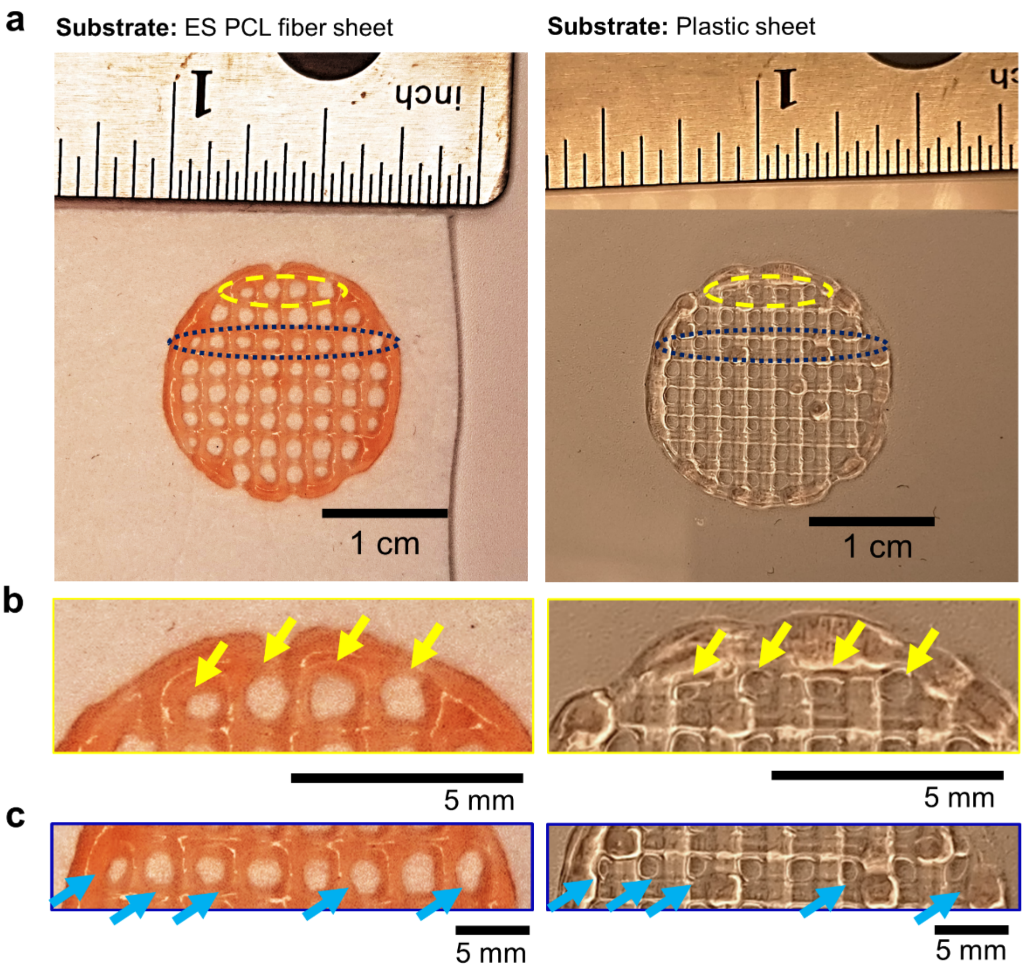

Figure S5. Low viscosity (5%) GelMA ink 3DB printing fidelity comparison (a) between AJ-3D ES PCL fiber sheet (left) and plastic substrates (right). b & c) High magnification images of yellow and dark blue dotted circles in (a). Yellow and blue arrows in (b & c) points the gaps between the square grids.

Supporting Videos

- **Video S1:** Flexibility and shape memory of the 3D human face anatomical PCL fiber scaffold by the AJ-3D electrospinning technique (MP4)
- **Video S2:** SA-dipcoated face construct demonstrating pliability and resiliency, mimicking native skin characteristics (MP4)
- **Video S3:** 3D depth profile analysis video showing the cell morphology and distribution across SA-dipcoated hybrid construct after 5 days into culture (MP4)
- **Video S4:** Monolayer of MDCK and lumenogenesis in the SA dipcoated hybrid construct after 35 days into culture (MP4)
- **Video S5:** GelMA hydrogel stability testing via bending and peel-off post-printing onto AJ-3D ES fiber sheet (MP4)
- **Video S6:** Structural integrity and shape memory of pocket casted SA hydrogel (MP4)
- **Video S7:** Freeform pocket 3DB printing into Carbopol-filled dome-shaped 3D PCL pocket using cell ink 3D bioprinter (MP4)
